# Benchmarking the Confidence of Large Language Models in Answering Clinical Questions: Cross-Sectional Evaluation Study

**DOI:** 10.2196/66917

**Published:** 2025-05-16

**Authors:** Mahmud Omar, Reem Agbareia, Benjamin S Glicksberg, Girish N Nadkarni, Eyal Klang

**Affiliations:** 1 Division of Data-Driven and Digital Medicine (D3M), Department of Medicine, Icahn School of Medicine at Mount Sinai, Gustave L. Levy Place New York, New York, NY, 10029, United States, 1 212 241 6500; 2 Ophthalmology Department, Hadassah Medical Center, Jerusalem, Israel

**Keywords:** safe AI, artificial intelligence, AI, algorithm, large language model, LLM, natural language processing, NLP, deep learning

## Abstract

**Background:**

The capabilities of large language models (LLMs) to self-assess their own confidence in answering questions within the biomedical realm remain underexplored.

**Objective:**

This study evaluates the confidence levels of 12 LLMs across 5 medical specialties to assess LLMs’ ability to accurately judge their own responses.

**Methods:**

We used 1965 multiple-choice questions that assessed clinical knowledge in the following areas: internal medicine, obstetrics and gynecology, psychiatry, pediatrics, and general surgery. Models were prompted to provide answers and to also provide their confidence for the correct answers (score: range 0%‐100%). We calculated the correlation between each model’s mean confidence score for correct answers and the overall accuracy of each model across all questions. The confidence scores for correct and incorrect answers were also analyzed to determine the mean difference in confidence, using 2-sample, 2-tailed *t* tests.

**Results:**

The correlation between the mean confidence scores for correct answers and model accuracy was inverse and statistically significant (*r*=−0.40; *P*=.001), indicating that worse-performing models exhibited paradoxically higher confidence. For instance, a top-performing model—GPT-4o—had a mean accuracy of 74% (SD 9.4%), with a mean confidence of 63% (SD 8.3%), whereas a low-performing model—Qwen2-7B—showed a mean accuracy of 46% (SD 10.5%) but a mean confidence of 76% (SD 11.7%). The mean difference in confidence between correct and incorrect responses was low for all models, ranging from 0.6% to 5.4%, with GPT-4o having the highest mean difference (5.4%, SD 2.3%; *P*=.003).

**Conclusions:**

Better-performing LLMs show more aligned overall confidence levels. However, even the most accurate models still show minimal variation in confidence between right and wrong answers. This may limit their safe use in clinical settings. Addressing overconfidence could involve refining calibration methods, performing domain-specific fine-tuning, and involving human oversight when decisions carry high risks. Further research is needed to improve these strategies before broader clinical adoption of LLMs.

## Introduction

With their capacity to understand and generate human-like text, large language models (LLMs) are poised to support health care professionals in complex clinical decisions [1-3]. A wide array of LLMs is now accessible, including open-source models, offering solutions that cater to both the public and medical professionals [1,4].

The efficacy of these models has been demonstrated in a variety of tasks, albeit with some limitations [5,6]. For instance, LLMs, such as GPT, have shown promise in providing diagnostic assistance and answering medical queries [5,7-9]. Katz et al [10] demonstrated that GPT-4 not only improved clinically when compared to its predecessor, GPT-3.5, but also matched physician performance in certain areas. However, there is evidence of hallucinations and inaccuracies in model outputs, which could lead to harm in clinical decision-making [11,12]. Specifically, LLMs have occasionally generated completely fabricated evidence (eg, information and references) and have presented such evidence as factual [11,12].

One way of building confidence in applying models within health care is the use of explainable artificial intelligence (AI) [13,14]. However, easily explainable outputs are difficult to evaluate due to the complexity of how LLMs process and output data [13,15,16]. Recent work revealed that these models often exhibit high confidence even when presenting incorrect information [17]. This raises questions about the underlying mechanisms that prompt an LLM to label certain statements as “more factual.” For example, one possible explanation could be that data-rich or frequently discussed topics in training sets may be perceived as more certain [18], even if this does not translate into clinical accuracy. Additionally, retrieval-augmented generation (RAG) has been proposed to ground LLM outputs in external data, which potentially mitigates hallucinations [19]. Nevertheless, these approaches do not fully resolve whether models can reliably judge their own correctness. Accurate and well-calibrated confidence scores may be vital for establishing trust in these systems, as such scores can alert users to approach certain responses with caution. If a model consistently shows undue confidence in wrong answers, it poses a subtle but potentially dangerous form of hallucination. Clinicians might adopt decisions based on erroneous advice that is delivered with overt certainty. By investigating how these models generate and express their confidence, we aimed to illuminate whether LLMs can reliably self-assess correctness.

The goal of this study was to benchmark LLMs (both proprietary LLMs, like GPT-4o and Claude 3.5 Sonnet, and open-source LLMs, like Qwen) in terms of accuracy and associated confidence in answering clinical questions. Our aim was to determine if these models can accurately judge when to be confident in their responses and, in doing so, allow for better explainability in their application.

## Methods

### Study Design and Data Source

This study used a public compiled dataset from a previous study by Katz et al [10], which includes 655 questions for the following five medical specialties: internal medicine, obstetrics and gynecology (OBGYN), psychiatry, pediatrics, and general surgery. These questions were sourced from official 2023 licensing examinations for each field and were crafted from internationally recognized textbooks and guidelines. This dataset serves as a standardized framework for assessment [20-24].

To enhance benchmarking reliability, each original question was rephrased twice by using the GPT-4 application programming interface (API) in Python (Python Software Foundation), yielding 1965 questions (we include the full prompt in Multimedia Appendix 1). The prompts were carefully designed to modify only the writing style, without altering any clinical details, such as medical terms, laboratory values, or answer choices [25]. This approach aimed to preserve all clinical details, ensuring that rephrased questions stayed faithful to the original intent and information. To confirm this, 2 board-certified physicians separately reviewed a 20% random sample of questions from each specialty. They compared the rephrased and original questions side by side, focusing on consistency in medical terminology, laboratory values, and answer choices. Both reviewers concluded that the paraphrased items remained unchanged in terms of clinical meaning and required no further edits, thereby confirming overall integrity and accuracy.

### Model Setup and Configuration

The LLMs used in this study were prompted (using 1 structured prompt) to return the correct answer, along with a confidence score for each choice (“A,” “B,” “C,” and “D”), in JSON format. These confidence scores were expressed as percentages between 0% and 100% for each option, resulting in a total confidence score of 100% for all options combined. The open access models were executed by using API codes in a dedicated server with 4 H100 80-GB graphics processing units; the corresponding codebase is accessible on GitHub for the original database by Katz et al [10], and we provide the full prompts, which can be used locally, in Multimedia Appendix 1. We used Python 3.10 for data analyses. The commercial models were used via the corresponding companies’ API interfaces. We used several Python libraries to facilitate data processing, model interaction, and analysis—NumPy 1.26.4, Pandas 2.1.4, Scikit-Learn 1.3.0, Hugging Face’s Transformers 4.37.2, and torch 2.2.2+cu121—as well as JSON module 2.0.9. We used the default hyperparameters for each model to reflect typical user settings and provide a balanced baseline [26]. For the open access models, we used the “instruct” versions, which perform better on zero-shot questioning.

### Benchmarked LLMs

**Figure 1.**
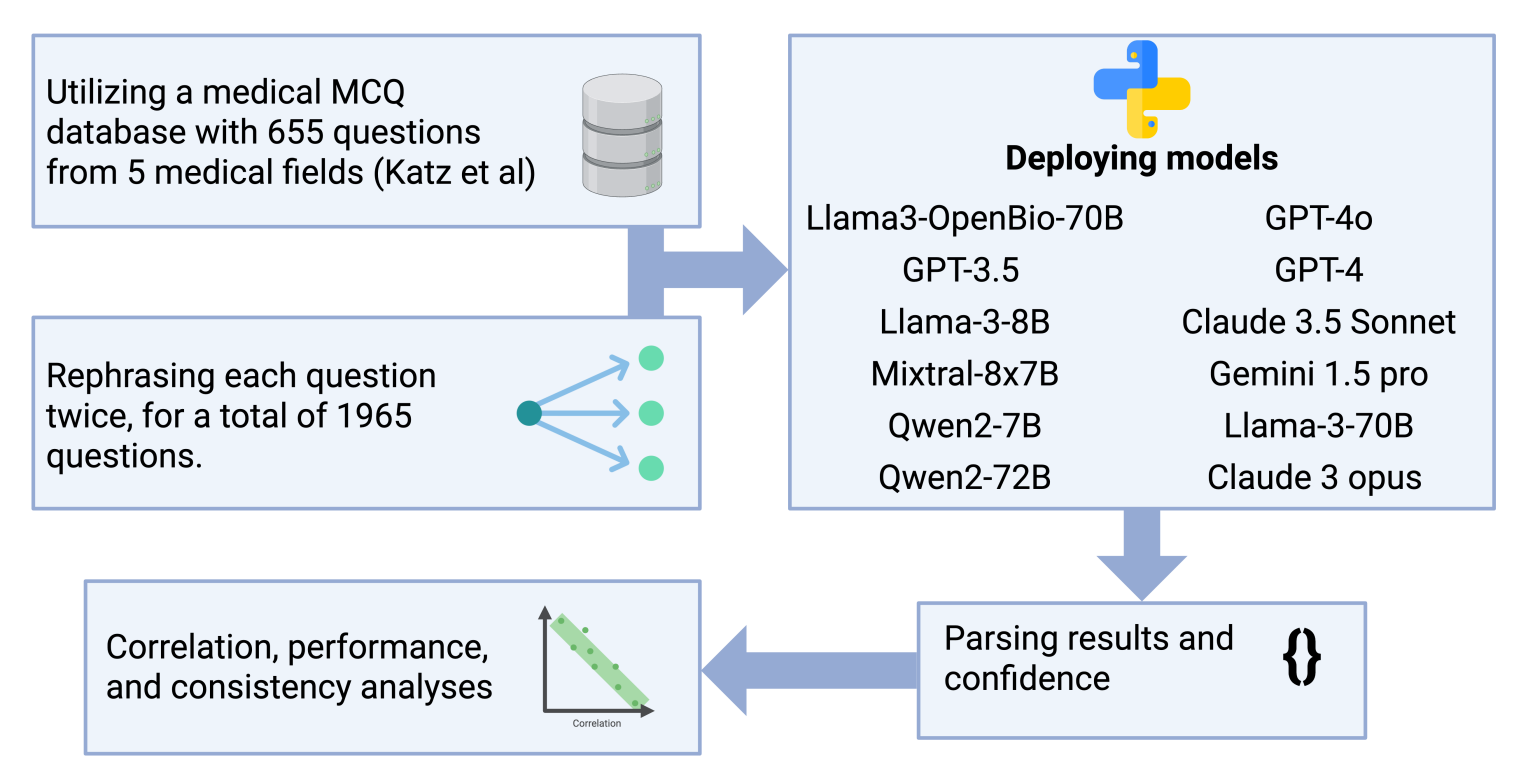
A flowchart representing the evaluation methodology. The 655 questions were sourced from a study by Katz et al [10]. MCQ: multiple-choice question.

We selected 12 LLMs that varied in terms of size, architectures, and intended domains (Figure 1). This set included established “household” models and newly introduced or domain-focused alternatives, ensuring diverse coverage (Figure 1). The benchmarked models are shown in Table S1 in Multimedia Appendix 1.

### Statistical Analysis

The Pearson correlation coefficient was used to correlate models’ mean confidence scores for correct answers and accuracies across models and medical fields. Chi-square tests assessed overall performance differences within each field, using proportions of correct responses. Post hoc pairwise comparisons with Bonferroni correction identified specific intermodel differences. Confidence levels were compared between correct and incorrect responses for each model, using 2-sample, 2-tailed *t* tests. Mean confidence scores were calculated for higher-tier and lower-tier models, as well as across all models. Performance consistency was evaluated by comparing confidence gaps between correct and incorrect responses. All statistical tests used a significance level of α=.05. Analyses were performed using R version 4.1.2 (R Foundation for Statistical Computing).

## Results

### Confidence Analysis

Table 1 summarizes accuracies and confidence levels across the models, and Table S2 in Multimedia Appendix 1 presents the data across all inspected fields and all models. An inverse correlation between the mean confidence scores for correct answers and the overall accuracy of the models is demonstrated (*r*=–0.40; *P*=.001); better-performing models generally showed lower confidence.

**Table 1.** Accuracies and confidence levels across the models.

Model	Accuracy, %	Total confidence, %	Confidence for correct answer, %	Confidence for incorrect answer, %
Claude 3.5 Sonnet	74	69.7	70.5	67.4
GPT-4o	73.8	63	64.4	59
Claude 3 Opus	71.7	68.5	68.9	67.3
GPT-4	66	84.1	84.5	83.3
Llama-3-70B	63.4	57.3	59.5	53.6
Llama OpenBio	59.2	77.9	77.7	78.1
Gemini	59.1	86.5	87.2	85.5
Qwen2-72B	57.8	57.7	58.6	56.5
Mixtral-8×7B	50.6	84.3	85.5	83
GPT-3.5	49	82.3	81.6	82.9
Llama-3-8B	48.4	80	79.7	80.3
Qwen2-7B	46	75.5	74.4	76.4

The mean confidence score for all 12 models was 76.1% when they were correct and 74.4% when they were incorrect. The 6 top-performing models showed a mean confidence score of 72.5% when they were correct and a mean confidence score of 69.4% when incorrect, while the 6 lowest-performing models displayed 79.6% confidence when they were correct and 79.5% confidence when they were incorrect (Table 2).

**Table 2.** Large language models’ mean confidence scores for correct and incorrect answers.

Model	Confidence when incorrect (%), mean (SD)	Confidence when correct (%), mean (SD)	*P* value
GPT-4o	58.99 (14.31)	64.38 (16.11)	.006
Llama-3-70B	53.59 (22.38)	59.50 (23.54)	.006
Claude 3.5 Sonnet	67.37 (9.08)	70.52 (11.07)	.003
Gemini	85.55 (16.23)	87.17 (16.58)	.35
Claude 3 Opus	67.32 (13.06)	68.90 (15.65)	.61
GPT-4	83.34 (23.30)	84.52 (22.43)	.07
Qwen2-72B	56.49 (18.55)	58.59 (20.03)	.004
Qwen2-7B	76.37 (17.11)	74.45 (20.30)	.01
Mixtral-8×7B	82.99 (16.52)	85.49 (14.62)	.04
Llama-3-8B	80.25 (17.40)	79.67 (21.59)	.31
Llama OpenBio	78.14 (27.59)	77.73 (28.78)	.83
GPT-3.5	82.85 (27.17)	81.63 (28.66)	.81

Four models (GPT-4o, Llama-3-70B, Claude 3.5 Sonnet, and Qwen2-72B) demonstrated significantly higher confidence when they were correct (all *P* values were <.01) across the different fields and subsets. Gemini exhibited the highest overall confidence levels (when incorrect: mean 85.6%, SD 16.2%; when correct: mean 87.2%, SD 16.6%). Qwen2-7B was unique in that it displayed higher confidence when incorrect (mean 76.4%, SD 17.1% vs mean 74.5%, SD 20.3% when correct; *P*=.01).

**Figure 2.**
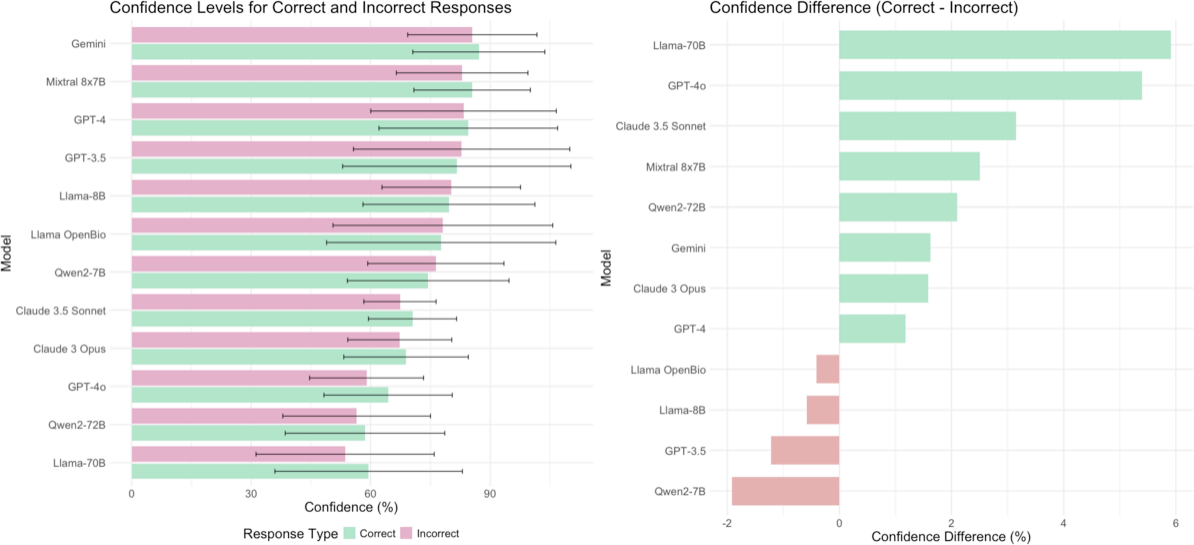
Large language models’ confidence results for correct and incorrect answers. The left graph displays the average confidence and 95% CIs for each model, categorized by correct answers (green) and incorrect answers (red). The right graph shows the differences in average confidence for each model, where green indicates higher confidence in correct answers, and red indicates higher confidence in incorrect answers.

GPT-3.5 and Llama-OpenBio-70B revealed minimal differences in confidence between correct and incorrect answers (*P*=.80). The largest confidence gap was observed in GPT-4 (5.4%, SD 2.3%; *P*=.003), while Llama-3-8B had the smallest gap (0.6%; Figure 2).

### Models’ Performances Across Fields

Significant differences were seen in model performance across all 5 medical specialties (at the *P*<.01 level). GPT-4o and Claude 3.5 Sonnet consistently outperformed other models. For internal medicine, GPT-4o (accuracy: 70.9%) and Claude 3.5 Sonnet (accuracy: 73.5%) showed no significant difference (*P*>.99) but outperformed lower-tier models, such as Qwen-7b (accuracy: 43.7%; *P*<.001). For OBGYN, Claude 3.5 Sonnet (accuracy: 71.0%) significantly outperformed most models, including GPT-4 (accuracy: 54.0%; *P*<.001). For pediatrics, the top 5 models (GPT-4o, Llama-3-70b, Claude 3.5 Sonnet, Claude 3 Opus, and GPT-4) showed no significant differences among themselves (all *P* values were >.05) but outperformed lower-tier models. Psychiatry results mirrored this pattern, with GPT-4o (accuracy: 84.4%) and Claude 3.5 Sonnet (accuracy: 82.4%) showing the best performance. For surgery, GPT-4o (accuracy: 70.9%) and Claude 3.5 Sonnet (accuracy: 70.5%) again showed no significant difference (*P*>.99) but outperformed lower-performing models, such as Qwen-7b (accuracy: 45.6%; *P*<.01; Tables S3 and S4 in Multimedia Appendix 1).

## Discussion

In our evaluation, accuracy and confidence were inversely correlated for LLMs. Some lower-complexity models were notably more confident in incorrect answers. Despite GPT-4o showing the best performance, its largest observed gap between confidence scores for correct and incorrect answers was only 5.4%. This indicates that it may be insufficient for reliably guiding clinical choices, although the difference was statistically significant, and the model’s confidence levels for correct and incorrect responses were generally high. Consequently, this gap does not provide a meaningful threshold for differentiating safe decision-making from potentially harmful decision-making in real-world practice. These results highlight potential risks in clinical applications, where model confidence, regardless of answer correctness, could lead to misinformed decisions.

We think that the observed miscalibration between correctness and confidence may pose risks in daily clinical practice if it remains unresolved. Overconfident models may recommend unsafe dosages or overlook key signs in a patient’s presentation, especially under the fast-paced pressures of modern practice. This could lead to incorrect prescriptions or treatments. For example, the model might prescribe an incorrect antibiotic for a resistant infection, thereby delaying proper care. In other cases, a model’s unwarranted confidence in a wrong triage decision could divert urgent attention from a critical patient. Such errors can increase morbidity and may undermine trust in AI-assisted clinical tools.

A brief comparison across models of various sizes did not reveal a consistent relationship between model size and confidence gaps. For instance, Qwen2-72B showed about a 2% difference in confidence between correct and incorrect responses, while Qwen2-7B exhibited a similarly small difference. This pattern was noted across multiple specialties, suggesting that architecture or domain-specific factors may play a more pivotal role than sheer model size in determining confidence behaviors.

Katz et al [10] reported that GPT-4 outperformed physicians in psychiatry and performed comparably to physicians in general surgery and internal medicine. Our study corroborates GPT-4’s strong performance, particularly in psychiatry, where GPT-4o achieved 84.4% accuracy. However, our findings suggest that more cautious interpretation is needed, given the high confidence levels observed for incorrect answers. Xiong et al’s [17] work on LLM confidence elicitation aligns with our observations of overconfidence. They noted improved calibration and failure prediction as model capability increased, which parallels our finding of better confidence calibration in more complex models.

If prompted confidence scores are truly driven by a model’s internal representations and are not random or uncontextualized outputs, then consistently arbitrary numbers would suggest a disconnect between the model’s knowledge state and its confidence estimates. Such misalignment can arise if the model’s architecture, training data, or prompting strategies do not calibrate confidence with genuine certainty [17]. In other words, a system might systematically generate high confidence, regardless of accuracy, if it lacks mechanisms or fine-tuning for self-regulating uncertainty [27]. Even larger models sometimes yield small or inconsistent confidence gaps, indicating that domain-specific refinements or improved calibration may be required. Without such refinements, confidence levels may remain weakly tied to actual reasoning processes, meaning that they would not reflect well-grounded internal assessments.

The implications for clinical practice warrant careful consideration. Although the performance leap of newer models is promising, their inability to accurately self-assess confidence across wrong answers poses risks. Two possible strategies for addressing these challenges can be the use of human-in-the-loop protocols and the implementation of ensemble methods [28].

Human-AI collaboration may offer a balanced approach to leveraging AI strengths while maintaining necessary human oversight in health care [29]. Sezgin [29] suggested a human-in-the-loop approach for ensuring that AI systems are supervised via human expertise. However, the effective implementation of this approach faces challenges. The careful design of user interfaces is important for preventing automation bias [29,30]. There are also concerns about the potential erosion of clinical skills as a result of overreliance on AI [31].

Emerging evidence also suggests that some prompt engineering techniques can reduce but not completely eliminate sociodemographic bias in model outputs [32]. However, studies continue to reveal significant sociodemographic biases in LLMs, such as a large-scale study by Omar et al [33]. These biases may affect patient prioritization, treatment recommendations, and mental health screening across different groups, potentially driving disparities in care [33]. Simply removing demographic variables (eg, gender and race) may also risk overlooking clinically relevant distinctions. In the context of our study, better-calibrated confidence outputs may help to mitigate such biases by allowing models to reliably signal uncertainty, which is especially important for sensitive medical decisions. Nonetheless, the comprehensive evaluation of these strategies requires longitudinal studies that monitor the evolution of biases and large-scale, globally diverse datasets, which can be used to refine mitigation approaches.

Ensemble methods, which aggregate multiple models, present another possible strategy [34]. Mahajan et al [35] conducted a review of ensemble learning techniques for disease prediction. They found that stacking—an ensemble method that combines multiple classifiers—showed the most accurate performance in 19 out of 23 cases. The voting approach was identified as the second-best ensemble method. However, ensemble methods are computationally intensive and may introduce latency in real-time clinical applications [36]. In some scenarios, a slight increase in overall accuracy might justify extra processing time, yet in urgent applications (eg, emergency triage), even brief delays can be problematic. Ensemble methods aggregate outputs from multiple models, distributing the “confidence load” so that individual sources of skewed certainty are less influential. However, our findings suggest that many current models show miscalibrated confidence levels. If all component models in an ensemble are prone to the same calibration issues, combining them may amplify rather than correct erroneous certainty.

Both strategies—human-in-the-loop protocols and the implementation of ensemble methods—would require extensive clinical trials for validation and the development of model-specific calibration curves for each medical specialty.

Our study has several limitations. The dataset was limited to 1965 multiple-choice questions for 5 medical specialties; therefore, the dataset may not fully represent the breadth of clinical scenarios. Further, the combination of automatic rephrasing and manual validation could have introduced bias [25]. We also used default model hyperparameters, which potentially limited performance optimization. To address these constraints, future work could expand the question sets (eg, by including a broader array of medical domains) and adopt real-world clinical data rather than purely examination-style questions. Additionally, custom hyperparameter tuning or advanced methods, such as RAG and fine-tuning, could be used to further refine model accuracy and confidence calibration [37], as the use of default hyperparameters, which may have varied across the evaluated LLMs, could have influenced their reported confidence levels. Finally, investigating computational cost and the time efficiency of deploying these models in clinical workflows would help to clarify practical feasibility.

In conclusion, better-performing LLMs show more aligned overall confidence levels, yet even the most accurate models still display minimal variation between right and wrong answers. This highlights a limitation in current self-assessment mechanisms and calls for further research. Future efforts could include larger and more diverse clinical datasets, domain-specific calibration strategies, and real-world testing to refine confidence estimates. Such work is critical before broader implementation of LLMs in clinical settings.

## Supplementary material

10.2196/66917 Multimedia Appendix 1 Supplementary materials with further information on the benchmarked large language models, their performance across different fields and specialties, and the prompt used for rephrasing the questions.

## Abbreviations

AI: artificial intelligence
API: application programming interface
LLM: large language model
OBGYN: obstetrics and gynecology
RAG: retrieval-augmented generation

## References

[1] Thirunavukarasu AJ, Ting DSJ, Elangovan K, Gutierrez L, Tan TF, Ting DSW (2023). Large language models in medicine. Nat Med.

[2] Clusmann J, Kolbinger FR, Muti HS (2023). The future landscape of large language models in medicine. Commun Med (Lond).

[3] Tayebi Arasteh S, Han T, Lotfinia M (2024). Large language models streamline automated machine learning for clinical studies. Nat Commun.

[4] Shah NH, Entwistle D, Pfeffer MA (2023). Creation and adoption of large language models in medicine. JAMA.

[5] Kanjee Z, Crowe B, Rodman A (2023). Accuracy of a generative artificial intelligence model in a complex diagnostic challenge. JAMA.

[6] Singhal K, Azizi S, Tu T (2023). Large language models encode clinical knowledge. Nature.

[7] Johnson D, Goodman R, Patrinely J (2023). Assessing the accuracy and reliability of AI-generated medical responses: an evaluation of the Chat-GPT model. Res Sq.

[8] Giannos P (2023). Evaluating the limits of AI in medical specialisation: ChatGPT’s performance on the UK Neurology Specialty Certificate Examination. BMJ Neurol Open.

[9] Hoch CC, Wollenberg B, Lüers JC (2023). ChatGPT’s quiz skills in different otolaryngology subspecialties: an analysis of 2576 single-choice and multiple-choice board certification preparation questions. Eur Arch Otorhinolaryngol.

[10] Katz U, Cohen E, Shachar E (2024). GPT versus resident physicians — a benchmark based on official board scores. NEJM AI.

[11] Omar M, Nassar S, Hijaze K, Glicksberg BS, Nadkarni GN, Klang E (2024). Generating credible referenced medical research: a comparative study of OpenAI’s Gpt-4 and Google’s Gemini. SSRN.

[12] Azamfirei R, Kudchadkar SR, Fackler J (2023). Large language models and the perils of their hallucinations. Crit Care.

[13] Yang G, Ye Q, Xia J (2022). Unbox the black-box for the medical explainable AI via multi-modal and multi-centre data fusion: a mini-review, two showcases and beyond. Inf Fusion.

[14] Soroush A, Glicksberg BS, Zimlichman E (2024). Large language models are poor medical coders — benchmarking of medical code querying. NEJM AI.

[15] Schwartz IS, Link KE, Daneshjou R, Cortés-Penfield N (2024). Black box warning: large language models and the future of infectious diseases consultation. Clin Infect Dis.

[16] Poon AIF, Sung JJY (2021). Opening the black box of AI-medicine. J Gastroenterol Hepatol.

[17] Xiong M, Hu Z, Lu X (2024). Can LLMs express their uncertainty? An empirical evaluation of confidence elicitation in LLMs. arXiv.

[18] Sarker IH (2021). Machine learning: algorithms, real-world applications and research directions. SN Comput Sci.

[19] Xiong G, Jin Q, Wang X, Zhang M, Lu Z, Zhang A (2025). Improving retrieval-augmented generation in medicine with iterative follow-up questions. Pac Symp Biocomput.

[20] Townsend CM, Beauchamp RD, Evers BM, Mattox KL (2016). Sabiston Textbook of Surgery: The Biological Basis of Modern Surgical Practice.

[21] Loscalzo J, Fauci AS, Kasper DL, Hauser SL, Longo DL, Jameson JL (2022). Harrison’s Principles of Internal Medicine.

[22] Kliegman RM, Behrman RE, Jenson HB, Stanton BMD (2007). Nelson Textbook of Pediatrics E-Book.

[23] American Psychiatric Association (2000). Diagnostic and Statistical Manual of Mental Disorders.

[24] Gabbe SG, Niebyl JR, Simpson JL (2016). Obstetrics: Normal and Problem Pregnancies E-Book.

[25] Soni S, Roberts K (2020). Paraphrasing to improve the performance of electronic health records question answering. AMIA Jt Summits Transl Sci Proc.

[26] Achiam J, Adler S, OpenAI (2024). GPT-4 technical report. arXiv.

[27] Liu L, Pan Y, Li X, Chen G (2024). Uncertainty estimation and quantification for llms: a simple supervised approach. arXiv.

[28] Longhurst CA, Singh K, Chopra A, Atreja A, Brownstein JS (2024). A call for artificial intelligence implementation science centers to evaluate clinical effectiveness. NEJM AI.

[29] Sezgin E (2023). Artificial intelligence in healthcare: complementing, not replacing, doctors and healthcare providers. Digit Health.

[30] Straw I (2020). The automation of bias in medical artificial intelligence (AI): decoding the past to create a better future. Artif Intell Med.

[31] Čartolovni A, Malešević A, Poslon L (2023). Critical analysis of the AI impact on the patient-physician relationship: a multi-stakeholder qualitative study. Digit Health.

[32] Omar M, Sorin V, Agbareia R (2024). Evaluating and addressing demographic disparities in medical large language models: a systematic review. medRxiv.

[33] Omar M, Soffer S, Agbareia R (2024). Socio-demographic biases in medical decision-making by large language models: a large-scale multi-model analysis. medRxiv.

[34] Yang H, Li M, Zhou H, Xiao Y, Fang Q, Zhang R (2023). One LLM is not enough: harnessing the power of ensemble learning for medical question answering. medRxiv.

[35] Mahajan P, Uddin S, Hajati F, Moni MA (2023). Ensemble learning for disease prediction: a review. Healthcare (Basel).

[36] Edeh MO, Dalal S, Dhaou IB (2022). Artificial intelligence-based ensemble learning model for prediction of hepatitis C disease. Front Public Health.

[37] Glicksberg BS, Timsina P, Patel D (2024). Evaluating the accuracy of a state-of-the-art large language model for prediction of admissions from the emergency room. J Am Med Inform Assoc.

