# Supplementary material for: Benchmarking the Confidence of Large Language Models in Answering Clinical Questions: Cross-Sectional Evaluation Study
Source: JMIR Med Inform. 2025 May 16;13:e66917. doi: 10.2196/66917 (PMC12101789; doi:10.2196/66917)
Supplement: Multimedia Appendix 1 [file medinform-v13-e66917-s001.docx]

**Table S1**: A list of the benchmarked LLMs.

| Model | Description |
| --- | --- |
| GPT-4 0613 and gpt-4o-2024-05-13 | Latest iterations of OpenAI's GPT series. |
| GPT-3.5 | Predecessor to GPT-4, still widely used in various applications. |
| Claude Sonnet 3.5 - 20240620 and Claude Opus 3- 20240229 | Recent models from Anthropic. |
| Gemini 1.5 Pro | Google's advanced model. |
| Llama-3-70B and Llama-3-8B | Different sizes of Meta's open-source Llama-3 series. |
| Llama3-OpenBio-70B | A domain-specific variant of the Llama model. |
| Mixtral-8x7B | An open-source mixture of experts (MOE) model developed by Mistral AI. |
| Qwen2-72B and Qwen2-7B | Different sizes of Alibaba's Qwen model series. |

**Table S2**

| Model | Field | Accuracy | Total Confidence | Correct Confidence | Incorrect Confidence |
| --- | --- | --- | --- | --- | --- |
| GPT4o | Surgery | 70.9 | 64.5 | 66.4 | 59.9 |
|  | OBGYN | 65.7 | 62.3 | 64.1 | 59 |
|  | Internal Medicine | 75.1 | 62.8 | 64.1 | 58.8 |
|  | Pediatrics | 71.7 | 64.6 | 66.8 | 59.1 |
|  | Psychiatry | 84.4 | 61.3 | 61.9 | 57.6 |
| Llama-3-70B | Surgery | 61.5 | 64.1 | 67.9 | 58 |
|  | OBGYN | 55.6 | 64.6 | 66 | 62.8 |
|  | Internal Medicine | 58.5 | 30.6 | 31.3 | 29.6 |
|  | Pediatrics | 68.4 | 65.4 | 67.2 | 61.5 |
|  | Psychiatry | 73.3 | 61.5 | 62.5 | 58.7 |
| Sonnet-3.5 | Surgery | 70.4 | 69.4 | 70.4 | 67.2 |
|  | OBGYN | 71 | 68.4 | 69.4 | 65.9 |
|  | Internal Medicine | 73.5 | 69 | 69.2 | 68.5 |
|  | Pediatrics | 71.4 | 70 | 71.3 | 66.8 |
|  | Psychiatry | 82.4 | 71.5 | 72 | 69.2 |
| Gemini | Surgery | 60.8 | 87.1 | 88.6 | 84.6 |
|  | OBGYN | 58.8 | 87.3 | 87.9 | 86.5 |
|  | Internal Medicine | 59 | 83.4 | 83.6 | 83.1 |
|  | Pediatrics | 57.6 | 88.6 | 88.7 | 88.4 |
|  | Psychiatry |  |  |  |  |
| Opus | Surgery | 67.1 | 70.6 | 71.9 | 68 |
|  | OBGYN | 71 | 68.4 | 69.4 | 65.9 |
|  | Internal Medicine | 71.7 | 68 | 68.2 | 67.6 |
|  | Pediatrics | 68 | 70.3 | 71.5 | 67.6 |
|  | Psychiatry | 79.1 | 65.6 | 65.1 | 67.5 |
| GPT-4 | Surgery | 66.7 | 86.2 | 86.3 | 86 |
|  | OBGYN | 54 | 86.5 | 84.8 | 88.5 |
|  | Internal Medicine | 64 | 83.4 | 85.7 | 79.4 |
|  | Pediatrics | 67 | 85.8 | 87.9 | 81.6 |
|  | Psychiatry | 77.3 | 79.4 | 80.2 | 76.8 |
| Qwen2-72B | Surgery | 57.4 | 59.5 | 60.5 | 58.2 |
|  | OBGYN | 55.2 | 59 | 57.5 | 60.8 |
|  | Internal Medicine | 48.1 | 57.3 | 58.6 | 56.1 |
|  | Pediatrics | 59.6 | 56.2 | 58.5 | 52.7 |
|  | Psychiatry | 67.3 | 56.1 | 57.9 | 52.5 |
| Qwen2-7B | Surgery | 45.6 | 76 | 73.8 | 77.8 |
|  | OBGYN | 43.6 | 78.1 | 76.2 | 79.6 |
|  | Internal Medicine | 43.9 | 77.5 | 79.4 | 76 |
|  | Pediatrics | 39.7 | 77.1 | 75.7 | 78 |
|  | Psychiatry | 54.2 | 69.9 | 69.7 | 70.1 |
| mixtral_8x7B | Surgery | 46.8 | 84.9 | 85.5 | 84.3 |
|  | OBGYN | 44.4 | 83.9 | 84 | 83.8 |
|  | Internal Medicine | 45.2 | 84.8 | 85.8 | 84 |
|  | Pediatrics | 52.2 | 84.1 | 85.7 | 82.2 |
|  | Psychiatry | 63.3 | 83.7 | 86.1 | 79.5 |
| Llama-3-8B | Surgery | 47.5 | 82.5 | 82.5 | 82.5 |
|  | OBGYN | 43.2 | 80.2 | 80.1 | 80.4 |
|  | Internal Medicine | 40.5 | 81.8 | 82.4 | 81.4 |
|  | Pediatrics | 48.5 | 84.6 | 86.3 | 83.1 |
|  | Psychiatry | 60.9 | 72.7 | 72.3 | 73.4 |
| Llama_openbio | Surgery | 56 | 78.1 | 79.7 | 76.2 |
|  | OBGYN | 53.7 | 80.8 | 79.9 | 81.9 |
|  | Internal Medicine | 54 | 77.9 | 78.2 | 77.6 |
|  | Pediatrics | 61.3 | 80.6 | 79.9 | 81.7 |
|  | Psychiatry | 70.4 | 73.2 | 73.2 | 73 |
| GPT-3.5 | Surgery | 48.7 | 86.3 | 86.2 | 86.4 |
|  | OBGYN | 42.9 | 83.4 | 83.5 | 83.2 |
|  | Internal Medicine | 45.8 | 84.7 | 85 | 84.5 |
|  | Pediatrics | 49.2 | 89.2 | 88 | 90.5 |
|  | Psychiatry | 57.6 | 70.7 | 70.9 | 70.6 |

**Table S3**: Confidence means of LLMs between correct and incorrect answers.

| **Model** | **Confidence When Incorrect (mean, SD)** | **Confidence When Correct (mean, SD)** | **p-value** |
| --- | --- | --- | --- |
| GPT-4o | 58.99 ± 14.31 | 64.38 ± 16.11 | <0.01 |
| Llama-3-70B | 53.59 ± 22.38 | 59.50 ± 23.54 | <0.01 |
| Claude 3.5 Sonnet | 67.37 ± 9.08 | 70.52 ± 11.07 | <0.01 |
| Gemini | 85.55 ± 16.23 | 87.17 ± 16.58 | 0.35 |
| Claude 3 Opus | 67.32 ± 13.06 | 68.90 ± 15.65 | 0.61 |
| GPT-4 | 83.34 ± 23.30 | 84.52 ± 22.43 | 0.07 |
| Qwen2-72B | 56.49 ± 18.55 | 58.59 ± 20.03 | <0.01 |
| Qwen2-7B | 76.37 ± 17.11 | 74.45 ± 20.30 | 0.01 |
| Mixtral 8x7B | 82.99 ± 16.52 | 85.49 ± 14.62 | 0.04 |
| Llama-3-8B | 80.25 ± 17.40 | 79.67 ± 21.59 | 0.31 |
| Llama OpenBio | 78.14 ± 27.59 | 77.73 ± 28.78 | 0.83 |
| GPT-3.5 | 82.85 ± 27.17 | 81.63 ± 28.66 | 0.81 |

**Table S4**: Top-performing models benchmarking results across different specialties.

| **Specialty** | **Model** | **GPT-4** | **Llama3-70b** | **Claude 3.5 Sonnet** | **Gemini 1.5 Pro** | **Claude 3 Opus** | **GPT-4** |
| --- | --- | --- | --- | --- | --- | --- | --- |
| Surgery | Overall | 70.92 | 61.47 | 70.45 | 60.76 | 67.14 | 69.50 |
|  | Original | 70.92 | 55.32 | 66.67 | 58.87 | 65.96 | 68.79 |
|  | Rephrase 1 | 68.79 | 65.25 | 68.79 | 59.57 | 67.38 | 68.79 |
|  | Rephrase 2 | 73.05 | 63.83 | 75.89 | 63.83 | 68.09 | 70.92 |
| Internal Medicine | Overall | 75.13 | 58.47 | 73.54 | 58.99 | 70.45 | 64.02 |
|  | Original | 73.81 | 57.14 | 71.43 | 56.35 | 66.67 | 61.11 |
|  | Rephrase 1 | 74.60 | 59.52 | 73.81 | 63.49 | 68.79 | 66.67 |
|  | Rephrase 2 | 76.98 | 58.73 | 75.40 | 57.14 | 75.89 | 64.29 |
| Obstetrics and Gynecology | Overall | 65.71 | 55.64 | 70.98 | 58.75 | 65.47 | 53.96 |
|  | Original | 69.06 | 53.96 | 72.66 | 57.55 | 68.35 | 56.12 |
|  | Rephrase 1 | 62.59 | 56.12 | 71.22 | 59.71 | 68.35 | 53.96 |
|  | Rephrase 2 | 65.47 | 56.83 | 69.06 | 58.99 | 59.71 | 51.80 |
| Pediatrics | Overall | 71.72 | 68.35 | 71.38 | 57.58 | 68.01 | 67.00 |
|  | Original | 68.69 | 64.65 | 70.71 | 54.55 | 67.68 | 64.65 |
|  | Rephrase 1 | 72.73 | 70.71 | 70.71 | 58.59 | 71.72 | 66.67 |
|  | Rephrase 2 | 73.74 | 69.70 | 72.73 | 59.60 | 64.65 | 69.70 |
| Psychiatry | Overall | 84.44 | 73.56 | 82.44 | NA | 79.78 | 77.33 |
|  | Original | 77.33 | 67.33 | 83.33 | NA | 77.33 | 78.00 |
|  | Rephrase 1 | 88.00 | 76.00 | 81.33 | NA | 81.33 | 77.33 |
|  | Rephrase 2 | 88.00 | 77.33 | 82.67 | NA | 80.67 | 76.67 |

**Table S5**: Lower-performing models benchmarking results across different specialties.

| **Specialty** | **Model** | **Qwen-2-72b** | **Qwen-7b** | **Mistral** | **Llama 8b** | **Llama Bio** | **GPT-3.5** |
| --- | --- | --- | --- | --- | --- | --- | --- |
| Surgery | Overall | 57.45 | 45.63 | 46.81 | 47.52 | 56.03 | 53.43 |
|  | Original | 53.90 | 46.10 | 45.39 | 46.81 | 58.87 | 52.48 |
|  | Rephrase 1 | 61.70 | 47.52 | 46.10 | 48.23 | 54.61 | 53.90 |
|  | Rephrase 2 | 56.74 | 43.26 | 48.94 | 47.52 | 54.61 | 53.90 |
| Internal Medicine | Overall | 47.88 | 43.65 | 44.97 | 40.48 | 53.70 | 45.77 |
|  | Original | 46.03 | 45.24 | 46.83 | 40.48 | 55.56 | 46.83 |
|  | Rephrase 1 | 49.21 | 42.86 | 43.65 | 40.48 | 52.38 | 43.65 |
|  | Rephrase 2 | 48.41 | 42.86 | 44.44 | 40.48 | 53.17 | 46.83 |
| Obstetrics and Gynecology | Overall | 55.16 | 43.65 | 44.36 | 43.17 | 53.72 | 42.93 |
|  | Original | 56.83 | 43.17 | 43.88 | 44.60 | 56.83 | 46.04 |
|  | Rephrase 1 | 55.40 | 43.88 | 46.04 | 39.57 | 50.36 | 41.01 |
|  | Rephrase 2 | 53.24 | 43.88 | 43.17 | 45.32 | 53.96 | 41.73 |
| Pediatrics | Overall | 59.60 | 39.73 | 52.19 | 48.48 | 61.28 | 49.16 |
|  | Original | 52.53 | 36.36 | 47.47 | 44.44 | 60.61 | 49.49 |
|  | Rephrase 1 | 61.62 | 45.45 | 53.54 | 51.52 | 62.63 | 46.46 |
|  | Rephrase 2 | 64.65 | 37.37 | 55.56 | 49.49 | 60.61 | 51.52 |
| Psychiatry | Overall | 67.33 | 54.22 | 63.33 | 60.89 | 70.44 | 57.56 |
|  | Original | 67.33 | 54.00 | 62.00 | 60.00 | 67.33 | 62.67 |
|  | Rephrase 1 | 65.33 | 53.33 | 62.67 | 60.00 | 72.00 | 51.33 |
|  | Rephrase 2 | 69.33 | 55.33 | 65.33 | 62.67 | 72.00 | 58.67 |

The prompt used for rephrasing the questions.

def rephrase_question_and_answers(question, answers, correct_answers):

    """

    Rephrase the question and choices without altering:

      - Medical or technical terms (e.g., "CABG," "PSA," "creatinine," "anaerobic coverage," etc.)

      - Critical lab data or numeric values (e.g., "BP 120/80 mmHg," "HbA1c 7.2%," etc.)

      - The underlying medical knowledge and meaning needed to solve the question

    The model should only rephrase the language, keeping these points unchanged:

      - The main context (e.g., the scenario, the relevant lab results)

      - All key medical or scientific information

      - The correct answer itself

    Return the result as a JSON-formatted string:

      {

        "question": <rephrased question>,

        "answers": {

          "A": <rephrased choice A>,

          "B": <rephrased choice B>,

          ...

        },

        "solution": <capital letter indicating correct choice>

      }

    """

    prompt_question = f"""

Please rephrase the MCQ question and its choices below, ensuring:

- You keep the same core meaning, medical knowledge, lab values, and terminology.

- You do not change essential medical words, abbreviations, or data.

- You only rephrase the language if it does not alter the necessary medical content.

Question:

{question}

Choices:

{answers}

Correct Answer(s):

{correct_answers}

When you respond, format your answer in strict JSON as follows:

{{

  "question": "<Revised question text>",

  "answers": {{

    "A": "<Revised choice A>",

    "B": "<Revised choice B>",

    "...": "..."

  }},

  "solution": "<Capital letter of the correct choice>"

}}

"""

    response_question = client.chat.completions.create(

        model="gpt-4o",

        messages=[

            {"role": "system", "content": "You are an expert medical doctor."},

            {"role": "user", "content": prompt_question}

        ],

    )

    rephrased_question = response_question.choices[0].message.content.strip()

    print(rephrased_question)

    return rephrased_question
